# Repeatability and Diagnostic Value of Nasal Potential Difference in a Genetically Admixed Population

**DOI:** 10.14740/jocmr2312w

**Published:** 2015-12-03

**Authors:** Izabela Rocha Sad, Laurinda Yoko Shinzato Higa, Teresinha Leal, Raisa da Silva Martins, Ana Claudia de Almeida, Eloane Goncalves Ramos, Giselda Maria Kalil de Cabello, Maria Virginia Marques Peixoto

**Affiliations:** a Pediatric Pulmonology, Pediatric Department, Fernandes Figueira National Institute of Woman, Child and Adolescent Health, Oswaldo Cruz Foundation/FIOCRUZ, Rio de Janeiro, Brazil; b Louvain Center for Toxicology and Applied Pharmacology (LTAP), Institut de Recherche Experimentale et Clinique (IREC) Universite Catholique de Louvain, Brussels, Belgium; c Laboratory of Quantitative Methods, Fernandes Figueira National Institute of Woman, Child and Adolescent Health, Oswaldo Cruz Foundation/FIOCRUZ, Rio de Janeiro, Brazil; d Clinical Research Unit, Fernandes Figueira National Institute of Woman, Child and Adolescent Health, Oswaldo Cruz Foundation/FIOCRUZ, Rio de Janeiro, Brazil; e Laboratory of Human Genetics, Oswaldo Cruz Institute, Oswaldo Cruz Foundation/FIOCRUZ, Pavilhao Leonidas Deane, sala 611, Rio de Janeiro, Brazil

**Keywords:** Cystic fibrosis, Nasal potential difference, Diagnostic test, Repeatability, Wilschanski index, Sermet score

## Abstract

**Background:**

The genetic diversity of the Brazilian population results from three ethnic groups admixture: Europeans, Africans and Amerindians, thus increasing the difficulty of performing cystic fibrosis (CF) diagnosis. The nasal potential difference (NPD) evaluates the cystic fibrosis transmembrane conductance regulator (CFTR) and epithelial sodium channel (ENaC) activity. Despite being a useful CF diagnostic test and a biomarker of CFTR-modulator drugs, it is also highly operator dependent. Therefore, it may be difficult to get accurate results and to interpret them. Wilschanski and Sermet scores were proposed to address these issues. This study aimed to evaluate repeatability and diagnostic value of NPD parameters and Wilschanski and Sermet scores in a CF center in Rio de Janeiro.

**Methods:**

NPD was performed in 78 subjects. Maximal PD, amiloride response, total chloride response, and Wilschanski and Sermet scores were explored as means (confidence interval, CI). One-way ANOVA was used to compare mean differences and Scheffe test was used to pair-wise comparisons. Repeatability was evaluated by scatter and Bland-Altman plots. The Ethics Committee of the CF Center has approved the study protocol. Parents and adult participants signed an informed consent form.

**Results:**

Forty-eight healthy-volunteers, 19 non-CF and 11 CF patients were enrolled in this study. Significant differences were found when comparing CF patients’ NPD parameters to the other two groups (P = 0.000). Moreover, no significant differences were found when parameters from non-CF patients were compared with those from healthy volunteers (P > 0.05). The means of NPD parameters and diagnostic scores of each group were in concordance with disease/non-disease conditions. The repeatability data - Wilschanski and Sermet and NPD - allow NPD to be performed in this Brazilian CF Center.

**Conclusions:**

The present study gathered consistent data for Bland-Altman plots. The results of Wilschanski and Sermet diagnostic scores suggest that they were concordant with CF/non-CF conditions. More NPD tests should be performed in the Rio de Janeiro CF dynamic cohort to contribute to international NPD validation studies and to provide NPD as a biomarker in Brazil.

## Introduction

Over the last years, knowledge about cystic fibrosis (CF) had added significant technologic and scientific advances. Definitions and nomenclatures have been revisited [1] and new CF phenotypes have been recognized, e.g. cystic fibrosis transmembrane conductance regulator (CFTR)-related disorders [2] and CFTR-related metabolic syndrome [3]. Genotype-phenotype correlations are being reviewed by the clinical and functional translation of CFTR (CFTR2) [4].

There are an emerging number of non-classical or atypical phenotypes. Patients with such uncertain diagnoses represent a challenge [2, 5-7]. Besides, newborn screening has contributed to growing not only the number of new diagnoses [3], as well as the number of CF equivocal diagnoses [8].

Sweat chloride test, *CFTR* mutation analysis and CFTR bioassays are the core diagnostic tests currently used in CF clinic [1, 3]. Nasal potential difference (NPD) is the only *in vivo* test able to provide an evaluation of sodium and chloride transport via assessment of transepithelial bioelectric properties [9-11]. As a functional test, NPD also distinguishes individuals with non-classic forms of CF with abnormal CFTR function is suspected [9] but sweat test or *CFTR* mutation analysis are inconclusive from subjects with normal CFTR function [2].

CF patients are characterized by having hyperpolarization in basal conditions and increased response to amiloride, both reflecting the removal of the inhibitory effect of CFTR on epithelial sodium channel (ENaC). Moreover, the reduction or even the absence of response of the nasal mucosa to topic perfusion with chloride-free solution and with isoproterenol indicates loss of function of CFTR-mediated chloride transport [6, 11-14].

NPD has historically been recognized as a procedure requiring rigorous conduct to ensure consistent and valid results [15]. Skill and experience are required to achieve accuracy and repeatability with such delicate method as NPD. It is also important to establish normal NPD values and intra-subject variability for CF patients and non-CF controls, especially in a country like Brazil where ethnic composition results from Europeans, Africans and Amerindians admixture [16], and is believed to be distinct from Europe and North America. The distribution of *CFTR* mutations in Brazil is heterogeneous, with the presence of less prevalent CF mutations [17]. The F508del frequency is close to 48% [16, 18-22] and CF prevalence is estimated at 1:10,000 [22].

Upon recognizing the challenging aspects inherent to the diagnostic process, specially related to non-classic phenotypes, and considering the Brazilian singularity - economic contrast, large genetic diversity and the enhance in newborn screening diagnosis - this study was proposed. The hypothesis was that NPD could be performed in this admixtured population and results could provide its variability.

The present study aimed to evaluate the repeatability of NPD at a CF center in Rio de Janeiro, Brazil, as well as to evaluate the diagnostic value of NPD test, Wilschanski index and Sermet scores.

## Methods

The study was held between 2009 and 2010 at Fernandes Figueira National Institute of Woman, Child and Adolescent Healthy, the quaternary hospital in Rio de Janeiro where the CF Center is situated. The study accomplished the checklist of standards for the reporting of diagnostic accuracy studies (STARD) [23].

### Subjects

NPD measurements were performed in three groups of participants: 1) CF patients previously diagnosed according to consensus [1, 3] and regularly followed up, 2) non-CF patients recruited in the Pulmonology Outpatient Clinics and 3) healthy-volunteers, including 13 CF parents, obligated heterozygous. Exclusion criteria were pregnancy or lactation, cigarette smoking, acute upper respiratory tract infection in the last 4 weeks prior to the NPD test, nasal polyps or previous nasal surgery. At NPD measurements, CF patients were clinically stable with no respiratory symptoms other than those normally experienced neither in any exacerbation treatment nor in any long-term oxygen therapy.

### Clinical and laboratory assessment

Gender and age at NPD measurements were recorded for all participants. In the CF group, age at diagnosis and clinical data related to disease status were collected within 6 months of NPD: height and weight for age; lung function, assessed by standard spirometer (Jaeger Master Scope, v.4.65, Care Fusion Ltd) and expressed by the percentage of forced expiratory volume in first second (FEV_1_) predicted for age, gender and height [24]; pancreatic function, assessed by pancreatic enzymes replacement therapy (PERT) and chronic colonisation by *Pseudomonas aeruginosa* was assessed by Leed's criteria [25]. In CF patients, sweat chloride measurement by quantitative pilocarpine iontophoresis test (QPIT) [26] and coulometry quantitative chloride analyses were performed at diagnosis in the CF center. DNA analyses were performed by the Laboratory of Human Genetics at Oswaldo Cruz Institute/FIOCRUZ. The mutations were analyzed by distinct methodologies: F508del - heteroduplex analysis; N1303K and G542X - polymerase chain reaction - restriction fragment length polymorphism; S4X, R334W and P205S - single-strand conformation polymorphism and sequencing [20]; W1282X - multiplex PCR/reverse hybridization procedure [18]. Identified mutations were categorized into classes as previously described [27] and classified according to CFTR2 [4].

### NPD

NPD measurements were performed by a single operator previously trained in a qualified European CF Center (Universite de Louvain, Brussels), according to the technique described by Leal et al (2003) [28].

NPD values were recorded using a high-impedance voltmeter (Knick Portamess^®^, Elektronische Messgerate, Berlin, Germany) connected to two silver chloride electrodes (Ag/AgCl). The reference electrode was immersed in an electrocardiogram conductive cream (SignaElectrode Cream) diluted (1:1 v:v) with Ringer’s solution to build a bridge, and then it was placed on a lightly diamond-tip drill scarified skin area of ± 2 - 3 mm in the right forearm of the subject. The exploring electrode was inserted in the distal end of the first lumen of a no. 6 pediatric double lumen silicone Foley catheter, filled with the cream. The second lumen was used for sequential perfusions of isotonic buffered solutions at a constant rate of 3 mL/min. Solutions, set at pH 7.4, were filtered (Acrodisc Syringe filter 0.2 μm, PallCo, Ann Harbor, MI, USA), warmed and perfused for at least 3 min. Potential difference (PD) measurements were initially performed with the probe positioned at 3.0 cm, 2.0 cm, 1.5 cm, 1.0 cm and 0.5 cm from nasal anterior tip and then fixed at the most negative position on the nasal floor. During an initial phase, perfusion with Ringer’s solution was made to obtain basal values, and then 100 μM amiloride was added to inhibit ENaC activity. A third modified Ringer’s solution without chloride and with amiloride was used to promote a gradient favorable to chloride efflux. Addition of 10 μM isoproterenol to the chloride-free solution was applied to induce CFTR-dependent chloride efflux upon intracellular accumulation of cAMP. For quality control, electrodes offsets were done and skin PD was measured at the beginning and at the end of each NPD procedure. Electrode offsets under short-circuit conditions around zero and skin magnitude < -30 mV were considered acceptable. After finishing the measurements, the recorded data were transferred to a desktop computer through a dedicated Paraly SW105^®^ software [28] and then tracings were constructed using Excel^®^ software.

When performed more than once, tests were repeated within at least 1-week interval. The two most stable tracings from each subject were used for data analysis. Stability at the end of each phase, required to start the next phase, was considered as a change of < 1 mV at least for 30 s in the end of each NPD phase [1, 12]. A second reader, blind to the disease condition, checked the tracings.

**Figure 1:**
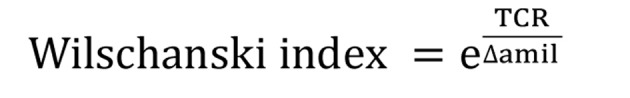
Wilschanski index.

**Figure 2:**
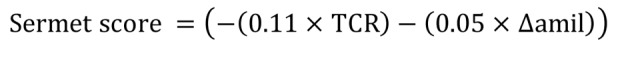
Sermet score.

Besides PDmax, representing the maximal basal PD value obtained at the end of perfusion with Ringer’s solution, the following NPD parameters and diagnostic index were recorded: 1) amiloride response (Δamil), representing the change observed in PD after perfusion with amiloride solution; 2) total chloride response (TCR), representing the sum of changes obtained after perfusion with chloride-free solution and with isoproterenol; 3) Wilschanski index (WI) [5] (Fig. 1); and 4) Sermet score (SS) [7] (Fig. 2).

In order to compare diagnostic scores, Δamil and TCR results from previous publications [5, 7, 11, 12, 28-32] were used to calculate WI and SS.

### Statistics

SPSS^®^22 Statistics Software was used for statistical analysis. Data were explored as means with standard deviation (SD) and confidence interval (CI), and median with range. One-way ANOVA was used to compare mean differences, and Levene’s test for variance homogeneity (data not shown). To pair-wise comparisons, the *post hoc* Scheffe test was performed. After checking normality of the distributions between group comparisons, they were evaluated by Kruskall-Wallis non-parametric method with *post hoc* comparisons being made pair-wise by Jonckheere-Terpstra. The null hypothesis was rejected at P < 0.05.

The first and second measurements were explored by scatter plot. The repeatability was evaluated by Bland-Altman plots [33], where the x-axis presents the average values of the first and second measurements and the y-axis presents the differences between them in order to show possible relationships between measurement error and true value. The precision was verified by SD and 95% CI for mean difference. The horizontal lines plotted denoted the average of the differences and the limits of agreement - mean difference ± 1.96 SD.

The Fernandes Figueira National Institute of Woman, Child and Adolescent Health’s Ethical Research Committee approved the study protocol and parents or participants gave written informed consent.

## Results

Seventy-eight subjects were enrolled in this study: CF patients (n = 11), seven males, with median age of 11.5 years (7.4 - 17.5); non-CF patients (n = 19), 10 males, with median age of 11.7 years (1.4 - 33.4) and healthy-volunteers (n = 48), 13 males, with median age of 32.4 years (21.8 - 60.3). Means (SD) values and respective 95% CI of all NPD parameters and diagnostic scores used for NPD interpretation are presented in Table 1. All of them showed significant differences between the groups (P < 0.000, one-way ANOVA). In pair-wise comparison, significant differences (P = 0.000, Scheffe test) were identified when CF group was compared to non-CF and to healthy-volunteers, and no significant differences were identified between non-CF and healthy-volunteers groups (P > 0.05, Scheffe test).

**Table 1:** Means (SD), 95% CI of NPD Parameters (mV), per Group and Pair-Wise Comparisons Between Groups

Groups (n)	PDmax^a^	Δamil^b^	TCR^c^	Wilschanski index^d^	Sermet score^e^
CF patients (11)	-31.7 (9.5) (-38.1 to -25.3)	15 (7.2) (10.2 to 19.8)	-1.5 (5.8) (-5.4 to 2.5)	0.9 (0.4) (0.7 to 1.2)	-0.6 (0.7) (-1.1 to -0.1)
Non-CF patients (19)	-16.4 (6.9) (-19.7 to -13.1)	7.5 (4.2) (5.5 to 9.5)	-15.3 (5.7) (-18.0 to -12.5)	0.1 (0.1) (0.1 to 0.2)	1.3 (0.6) (1.0 to 1.6)
Healthy-volunteers (48)	-15.2 (7.6) (-17.4 to -13.0)	7.8 (4.7) (6.5 to 9.2)	-13.0 (6.7) (-15 to -11.1)	0.2 (0.2) (0.2 to 0.3)	1.0 (0.7) (0.8 to 1.2)
One way ANOVA (P-value)	0.000	0.000	0.000	0.000	0.000
Pair-wise comparison	*Post hoc* Scheffe (P-value)				
CF × non-CF	0.000	0.001	0.000	0.000	0.000
CF × healthy-volunteers	0.000	0.000	0.000	0.000	0.000
Non-CF × healthy-volunteers	0.851	0.967	0.435	0.558	0.359

^a^PDmax: maximal basal PD; ^b^Δamil: change in PD after amiloride solution infusion; ^c^TCR: change in PD after zero chloride and isoproterenol solutions infusions; ^d^Wilschanski index = e^TCR/Δamil^; ^e^Sermet score = -(0.11 × TCR) - (0.05 × Δamil).

**Figure 3:**
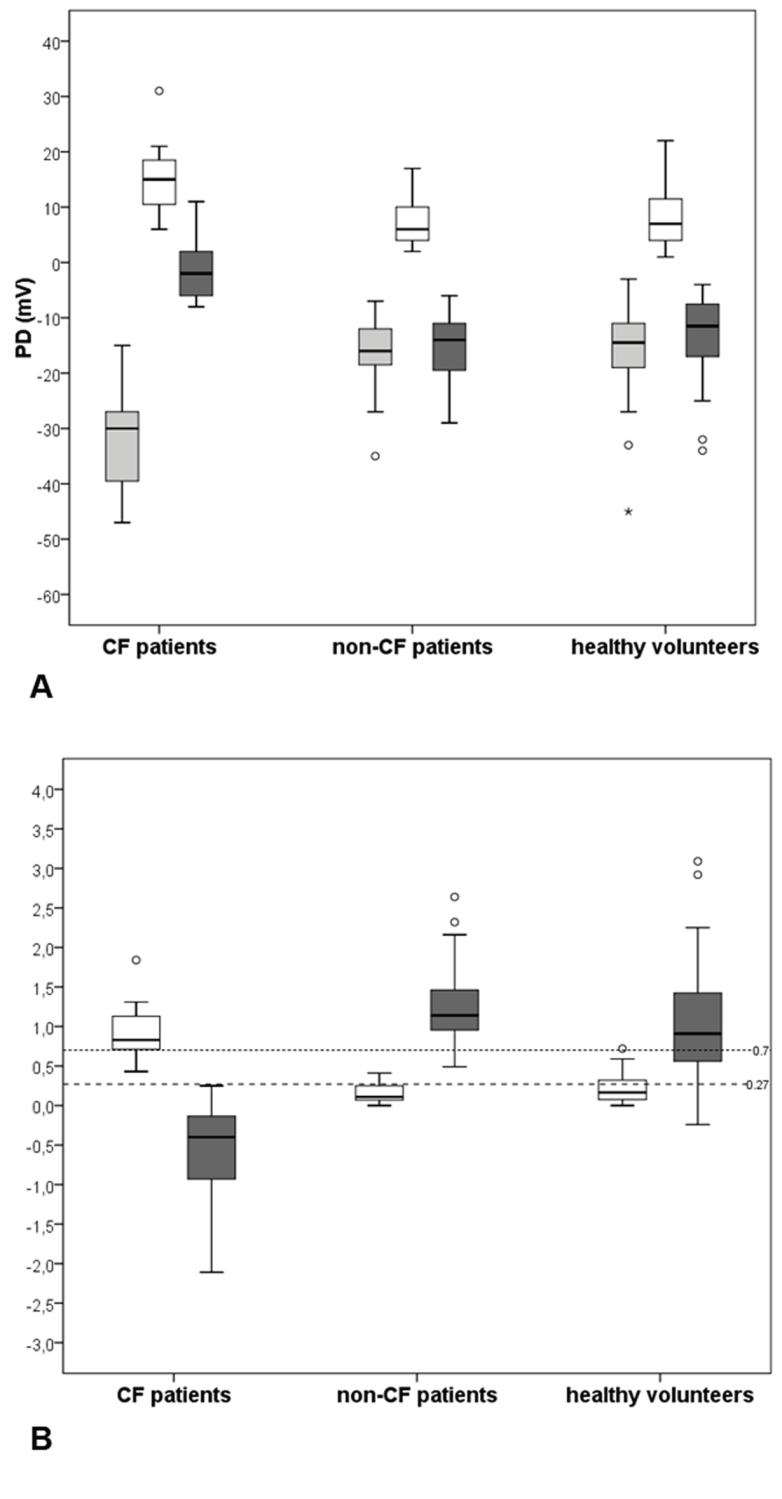
Medians and interquartile intervals of NPD parameters and diagnostic indices among groups. (A) Box plots of PDmax (gray boxes), amiloride response - Δamil (white boxes) and total chloride response - TCR (dark gray boxes). (B) Box plots of Wilschanski index (white boxes) and Sermet score (dark gray boxes) obtained from NPD measurements performed in 11 CF patients, 19 non-CF patients and in 48 healthy-volunteers. The horizontal black line across each box indicates the sample median of the corresponding group. Upper and lower horizontal box lines illustrate the 25th and the 75th percentiles; extreme upper and lower lines represent the 0.5th and the 99.5th percentiles of the variable. The small dashed horizontal line represents the cut-off value for CF diagnosis according to Wilschanski index (> 0.7) and the large dashed horizontal line represents the cut-off value for CF diagnosis according to Sermet score (≤ 0.27).

Medians values of NPD parameters and respective box plots among the three groups are shown in Figure 3A and Supplementary 1 (www.jocmr.org). In Figure 3B, the non-CF group box plots were completely outside the disease ranges. Among the 48 healthy-volunteers, there was one outlier just above the cut-off of WI (0.72) and four observations (from 0.13 to 0.24) were inside instead of outside the disease range of SS. In the CF group, the medians of both scores were inside CF disease ranges, but three patients had WI lying below and not above the predicting disease cut-off.

**Figure 4:**
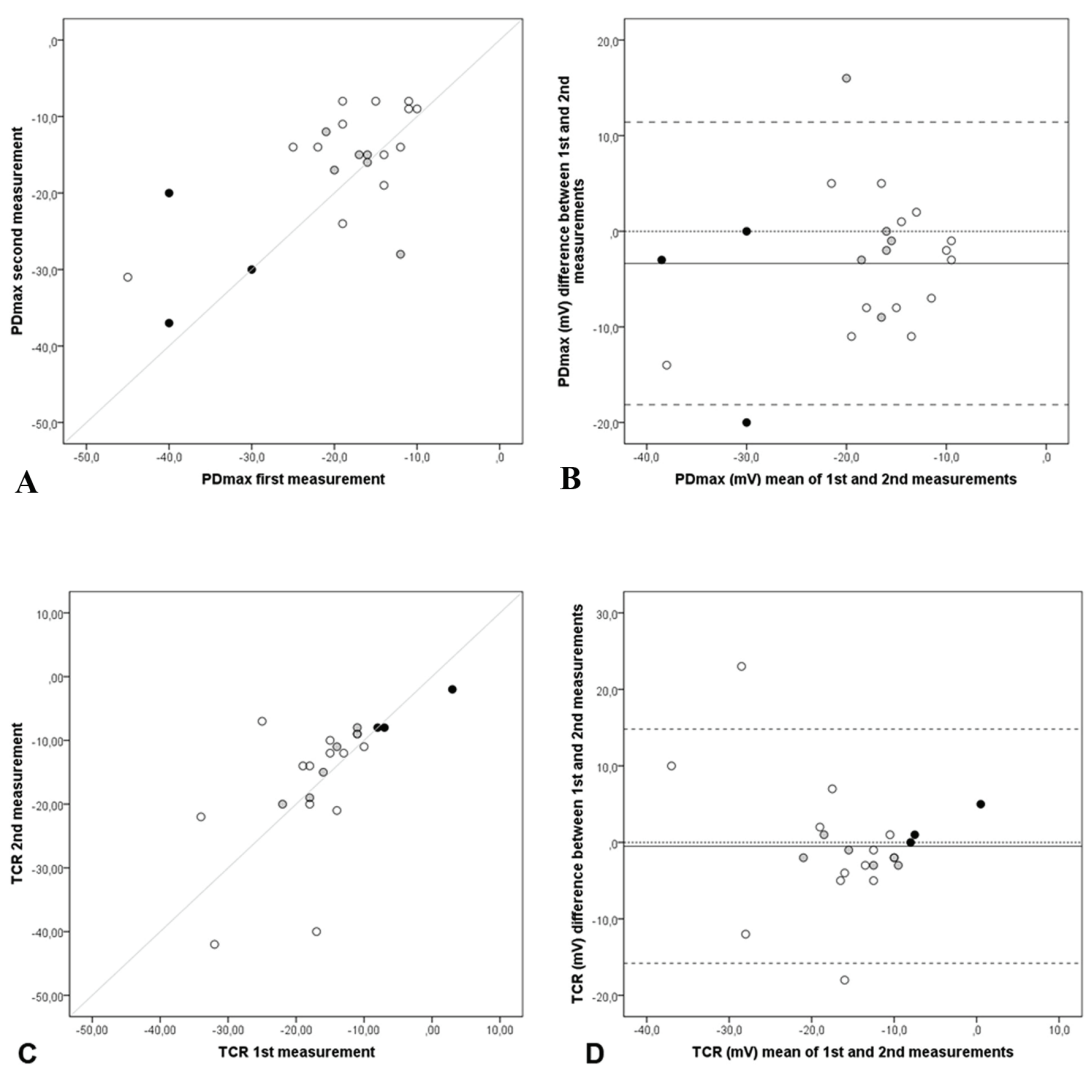
Repeatability of NPD parameters: PDmax and TCR. Scatter and Bland-Altman plots of first and second measurements: (A) scatter plot of PDmax, (B) Bland-Altman of PDmax, (C) scatter plot of TCR and (D) Bland-Altman of TCR of each subject (n = 22), CF patients (black dots), non-CF patients (grey dots) and healthy volunteers (white dots), with the horizontal lines representing the perfect agreement (dotted line), the value of the mean difference (small dashed line) and limits of agreement of 95% CI (large dashed lines).

Twenty-two participants repeated the exam. First and second measurements of PDmax and TCR are illustrated in Figure 4A and 4C. As they showed some differences, Bland-Altman plots were also used. In Figure 4B, the PDmax mean difference (PDmax_first_ - PDmax_second_) was -3.4 mV (SD = 7.5), 95% limits of agreement (11.4 to -18.1). Only two pair measurements were not inside the limits of agreements of ± 1.96 SD, one from the CF group and the other from the non-CF group. In Figure 4D, the TCR mean difference (TCR_first_ - TCR_second_) was -0.5 mV (SD = 7.81), 95% limits of agreement (14.80 to -15.80). Measurements observed outside the limits of agreement could be noticed twice in TCR Bland-Altman plot, both from healthy-volunteers group.

**Figure 5:**
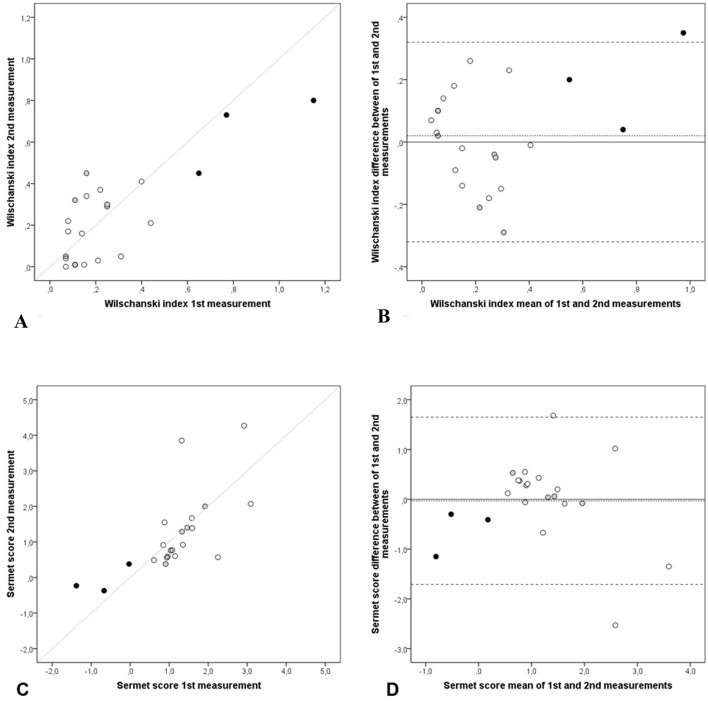
Repeatability of NPD parameters: NPD diagnostic indices: Wilschanski index and Sermet score. Scatter and Bland-Altman plots of first and second measurements: (A) scatter plot of Wilschanski index, (B) Bland-Altman of Wilschanski index, (C) scatter plot of Sermet score and (D) Bland-Altman of Sermet score of each subject (n = 22), CF patients (black dots), non-CF patients (grey dots) and healthy volunteers (white dots), with the horizontal lines representing the perfect agreement (dotted line), the value of the mean difference (small dashed line) and limits of agreement of 95% CI (large dashed lines).

In Figure 5B, the WI mean difference (WI_first_ - WI_second_) was 0.02 (SD = 0.16), 95% limits of agreement (0.32 to -0.32). In Figure 5D, the SS mean difference (SS_first_ - SS_second_) was -0.03 (SD = 0.86), 95% limits of agreement (1.65 to -1.71). All groups had observations inside respective limits of the agreement ± 1.96 SD, exception for three observations, one from CF patient in WI and two from healthy-volunteers in SS.

Data about diagnostic, clinical characteristics, disease severity and NPD results from all CF patients (n = 11) are presented in Table 2. In this group, the median age at diagnosis was 14 months (2 - 144). Patients with sweat chloride ≥ 60 mmol/L and severe CF-causing mutations accounted to the majority of the cohort. All patients had *Pseudomonas aeruginosa* infection, seven of them being chronically colonized according to Leed’s criteria, and more than 50% of patients had ventilator disturbance, with FEV_1_ below the predicted.

**Table 2:** Clinical Data Diagnosis, Disease Status and Nasal Potential Difference Results From CF Patients (n = 11)

	Sex	Diagnostic data				Disease status		NPD parameters and diagnostic indices				
		Age at diagnosis (months)	Sweat Cl^-^^a^ (mmol/L)	Genotype^b^	Class	FEV_1_^c^ (% pred)	Chronic Pa^d^	PDmax^e^ (mV)	Δamil^f^ (mV)	TCR^g^ (mV)	Wilschanski index^h^	Sermet score^i^
1	M	2	100.7	F508del/F508del	II/II	81.4	No	-47	16	11	1.84	-2.11
2	F	4	96.0	F508del/F508del	II/II	76.7	Yes	-20	6	-5	0.43	0.25
3	M	9	82.5	F508del/F508del	II/II	103.0	Yes	-25	7	-1	0.87	-0.24
4	F	5	88.5	F508del/F508del	II/II	77.4	Yes	-30	16	-7	0.65	-0.03
5	M	80	78.6	F508del/F508del	II/II	50.7	Yes	-34	11	-7	0.53	0.22
6	M	14	59.0	F508del/W1282X	II/I	116.2	No	-29	15	4	1.31	-1.19
7	F	3	98.0	N1303K/G542X	II/I	45.8	Yes	-39	11	-2	0.83	-0.33
8	M	72	55.3	S4X/R334W	I/IV or V	124.0	No	-37	25	-8	0.73	-0.37
9	M	86	79.3	F508del/NI	II/-	64.9	Yes	-15	10	1	1.11	-0.61
10	M	144	52.9	F508del/NI	II/-	98.3	Yes	-40	21	3	1.15	-1.38
11	F	33	77.4	P205S/NI	IV/-	64.0	No	-30	19	-5	0.77	-0.40

^a^QPIT sweat test; ^b^NI: not identified; ^c^FEV_1_ = forced expiratory volume in the first second; ^d^Pa: *Pseudomonas aeruginosa*; ^e^PDmax = maximum PD after Ringer’s solution perfusion; ^f^Δamil = PD changes after amiloride solution perfusion; ^g^TCR (total chloride response) = PD changes after perfusion with zero Cl^-^ and isoproterenol solutions; ^h^Wilschanski index: CF if > 0.7; ^i^Sermet score: CF if ≤ 0.27.

## Discussion

The repeatability data of this study were in line with the initial hypothesis that NPD could be performed in this Brazilian CF center because all NPD parameters analyzed - PDmax, Δamil and TCR - provided similar results to other CF centers [5, 7, 11, 12, 28-32, 34-36]. The WI and SS estimated in pair measures also confirmed its consistency.

All NPD were performed according to the modified technique proposed by Leal et al in 2003 [28]. In order to reduce variability, the same voltmeter, catheters and solutions were used. The sequence of solutions and duration of infusions were equally controlled. NPD measurements were well tolerated. Some subjects sneezed or complained about tickling sensations while the operator was searching for best position for the catheter in the nostrils.

The measurements were performed in the nostril with the largest basal PD according to Yaakov et al [32]. They have proposed that this nostril should be selected for drug and electrolyte responses, with the advantage of shortening the total duration of the protocol without missing data. Recent studies, however, suggested that both nostrils should be used [15, 30, 35]. There are still controversies about which values include in NPD analysis. Some authors use the average of both nostrils results while others prefer to use the results separately [9]. Some researchers use tracing values from the nostril with the largest TCR [35], while others prefer to use the side that provides technically better tracing [37]. In this study, the time period between repeated procedures was variable, what can support Yaakov’s statement that the NPD is repeatable regardless of the time between the measurements [32].

### NPD interpretation parameters: PDmax, Δamil and TCR

In normal airway epithelia and under basal conditions, sodium absorption is the primary ion transport activity and basal potential difference is negative [9]. In CF subjects, a hyperpolarized PDmax is observed reflecting enhanced sodium transport across a chloride-impermeable barrier [6], thought to be due to the absence of regulation of ENaC function by CFTR [9]. In both cases, it is expected for the nasal mucosa to depolarize during perfusion of amiloride, but in CF patients, Δamil is usually larger than in healthy subjects because of their initial hyperpolarization [34]. In non-CF subjects, a high total chloride response is expected. It is represented by a large hyperpolarization, explained by diffusion of chloride through ion channels after a low chloride extracellular and by CFTR-mediated chloride transport enhanced pharmacologically by the addition of isoproterenol. After perfusion with both chloride-free solutions, CF patients have TCR close to zero with small or no change in PD [5, 7, 12, 28-32, 34, 36]. TCR is the most important parameter for NPD interpretation because it is widely considered the most sensitive and specific indicator of the CFTR-dependent chloride transport, which reflects the activity of CFTR chloride channel [32, 38].

In this study, means and medians were used to summarize NPD parameters in order to enhance comparisons with other studies; however, some differences in techniques always need to be considered. The medians of PDmax and calculated parameters (Δamil and TCR) in non-CF and healthy-volunteers groups (Supplementary 1, www.jocmr.org) were similar to previous publications [7, 38]. Among CF patients, PDmax was smaller and Δamil was almost half of these publicated values [7, 38]. The means of PDmax, Δamil, TCR and diagnostic scores were significantly different between the three groups and the CF one was responsible for it (Table 1). The pair-wise comparison showed that PDmax was able to distinguish CF from non-CF and from healthy-volunteers. The values of PDmax obtained in CF were much closer to two studies [32, 34] than to others, in which basal hyperpolarization around -45 mV has been associated to CF patients [5, 7, 12, 28-31, 36]. As previously published [7, 11, 28, 30], in this study, the depolarization after amiloride was much larger in CF patients (Fig. 3A) and was able to discriminate them from other subjects (Table 1). The CF patients’ lack of response to perfusion with zero chloride plus beta-agonist solutions was evidenced (Table 1 and Fig. 4A). Similar TCR values -1.7 mV, -0.7 mV and 0.1 mV were observed in studies from the United Kingdom and the United States [31], from France [12] and from Australia [11], respectively. The repolarizations observed in both groups without CF (Fig. 3A and Table 1) were in accordance with some studies [12, 28, 31, 32] but different from other studies where TCR was much larger, around -25 mV [11, 29, 34-36].

### NPD diagnostic scores

In 1997, when Ho et al analyzed correlation between mutations types and clinical conditions in 61 normal and 22 CF subjects, they arbitrarily choose as representative of “low chloride secretors”, patients with TCR values ≤ 10 mV and as “high secretors”, patients with TCR values > 10 mV [36]. In 2008, Leal et al classified subjects as secretors when TCR < -5 mV and non-secretors when TCR > -5 mV [12]. In 2010, Middleton and House suggested that a PDmax value > 30 mV and a TCR < 10 mV would be a reasonable choice to define a distinction between CF and non-CF [11]. In the absence of widely acceptable NPD reference range, Ooi and Durie (2012) considered intermediate category for CFTR function. Therefore, they established: normal, when TCR < -12 mV; intermediate, when TCR results between -12 and -7.7 mV; and no function when TCR > -7.7 mV (between -7.7 and zero or positive) [39].

The need for accurate and reliable evaluation of CFTR function for diagnosis, management and consultation is broadly recognized [11, 39]. Neither the American nor the European Standard Operating Procedures establish how to interpret NPD results [37]. To better evaluate NPD measurements, it was important to provide indices that take into account both sodium and chloride transports. WI [5] and SS [7] distinguish CF patients with the following cut-offs: WI > 0.70 and SS ≤ 0.27. Nowadays, they are considered alternative derived endpoints and good models to discriminate between CF and healthy subjects, with reasonable diagnostic accuracy [9, 32].

The means of WI in the present study (Table 1) were inside the respective results ranges calculated in previous studies: between 0.85 and 1.11 in CF and between 0.03 and 0.32 in non-CF patients or healthy-volunteers (Supplementary 2, www.jocmr.org). The same happened to SS. The means of the present study and the ones from other authors lied in the range of respective group classification: from -2.08 to -0.81 for CF, and from 0.82 to 2.47 for non-CF or healthy-volunteers.

### Repeatability

The exploratory analysis of PDmax (Fig. 4A) and TCR (Fig. 4C) first and second measures initially took into account the scatter plots and the line of perfect agreement. All observations were dispersed around the line. Other authors [11, 30] have found similar results when they compared right and left nostrils of PDmax values. Aiming to increase NPD repeatability, Vermeulen et al [34] tested changes in the technique. Theirs repeated measures, founded with the side-hole catheter at most negative PD position at nasal floor, were suchlike the present study’s TCR repeated parameters (Fig. 4C). However, the best first × second TCR agreements were observed when the same authors used the larger surface catheter fixed at 5 cm on the nasal floor.

When examining repeatability in Bland-Altman plots, the ideal is that the line of mean differences be equal to zero, featuring a perfect agreement. If it is not, it is expected that all differences markers lie between the 95% limits of agreement of mean of difference. In this study, when data of all 22 subjects that repeated NPD were analyzed together, the mean (SD) of the differences between first and second PDmax was -3.4 (7.5), but it was -2.68 (7.1) for 19 controls separately (data not shown). The means (SD) of differences were smaller among others studies disease controls or healthy-volunteers: -1.4 (8.9) [32], -1.7 (7.2) [30] and -1.1 (4.7) [34], but in all of them, the variability shown by the SD remained equally high.

When non-CF and healthy-volunteers data (n = 19) were analyzed together, the mean (SD) of the differences between first and second TCR was 0.90 (8.33) with limits of agreement from -15.43 to 17.23. These results are in consonance with early works [30, 34, 35] that also analyzed NPD repeatability and found high variability with large limits of agreement.

In scatter plots of WI (Fig. 5A) and SS (Fig. 5C), first and second measurements are randomly dispersed around the line of perfect agreement, which can suggest the absence of bias. In the same direction, WI and SS means of differences in the Bland-Altman plots (Fig. 5B and 5D) were observed very close to zero: 0.02 and -0.03, respectively. In this study, only one from the 22 repeated measures of WI was beyond the 95% limits of agreement. This fact can be considered a satisfactory outcome when applying Bland-Altman plots. Furthermore, the random distribution of observations around zero suggests absence of bias in both graphs (Fig. 5B, D).

A single operator performing all NPD exams can be considered a strength factor of this study because it avoided inter-operator variability in a procedure considered very delicate and full of variability sources. Another strength of the present work is the possibility to confirm the applicability of WI and SS diagnostic indices with different previous publications data and to find concordant results to respective diseases status of all subjects groups. The quality of the repeatability results needs to be considered according to the NPD technique used in the present study. At this time NPD used to have less offsets steps and the standard operating initiatives were just beginning.

The small number of CF participants can be considered a weakness of the study. Fernandes Figueira National Institute of Woman, Child and Adolescent’s Healthy is a pediatric CF Center and the patient’s small age reduced the population eligibility as NPD measurement usually demands subject’s collaboration. Besides that, healthy-volunteers did not perform sweat test or genetic analysis as the absence of clinical symptoms was considered enough to include volunteers in this study. Only one nostril was analyzed and when this study was held, no standard operative procedure was available yet.

### Conclusion

The present study gathered consistent data between repeated measures for Bland-Altman plots. The results of Wilschanski and Sermet diagnostic scores suggest that they were concordant with CF/non-CF conditions. More NPD tests should be performed in the Rio de Janeiro CF dynamic cohort to contribute to international NPD validation studies and to provide NPD as a biomarker in Brazil.

## Abbreviations

CF: cystic fibrosis
CFTR: cystic fibrosis transmembrane conductance regulator
NPD: nasal potential difference
